# Identification, validation, and targeting of the mutant p53-PARP-MCM chromatin axis in triple negative breast cancer

**DOI:** 10.1038/s41523-016-0001-7

**Published:** 2017-01-19

**Authors:** Wei-Gang Qiu, Alla Polotskaia, Gu Xiao, Lia Di, Yuhan Zhao, Wenwei Hu, John Philip, Ronald C. Hendrickson, Jill Bargonetti

**Affiliations:** 1 The Department of Biological Sciences Hunter College, City University of New York, Hunter College-Weill Cornell Belfer Research Building, 413 East 69th, New York, NY 10065 USA; 2 The Graduate Center PhD Program in Biology, City University of New York, New York, NY 10016 USA; 3 Department of Physiology and Biophysics, Weill Cornell Medical College of Cornell University, New York, NY 10065 USA; 4 Rutgers Cancer Institute of New Jersey, New Brunswick, NJ 08903 USA; 5 Proteomics Core Facility, Memorial Sloan-Kettering Cancer Center, New York, NY 10065 USA; 6 The Graduate Center PhD Programs in Biology and Biochemistry, City University of New York, New York, NY 10016 USA; 7 Department of Cell and Developmental Biology, Weill Cornell Medical College of Cornell University, New York, NY 10065 USA

## Abstract

Over 80% of triple negative breast cancers express mutant p53. Mutant p53 often gains oncogenic function suggesting that triple negative breast cancers may be driven by p53 protein type. To determine the chromatin targets of this gain-of-function mutant p53 we used inducible knockdown of endogenous gain-of-function mtp53 in MDA-MB-468 cells in conjunction with stable isotope labeling with amino acids in cell culture and subcellular fractionation. We sequenced over 70,000 total peptides for each corresponding reciprocal data set and were able to identify 3010 unique cytoplasmic fraction proteins and 3403 unique chromatin fraction proteins. The present proteomics experiment corroborated our previous experiment-based results that poly ADP-ribose polymerase has a positive association with mutant p53 on the chromatin. Here, for the first time we report that the heterohexomeric minichromosome maintenance complex that participates in DNA replication initiation ranked as a high mutant p53-chromatin associated pathway. Enrichment analysis identified the minichromosome maintenance members 2–7. To validate this mutant p53- poly ADP-ribose polymerase-minichromosome maintenance functional axis, we experimentally depleted R273H mutant p53 and found a large reduction of the amount of minichromosome maintenance complex proteins on the chromatin. Furthermore a mutant p53-minichromosome maintenance 2 direct interaction was detected. Overexpressed mutant p53, but not wild type p53, showed a protein-protein interaction with minichromosome maintenance 2 and minichromosome maintenance 4. To target the mutant p53- poly ADP-ribose polymerase-minichromosome maintenance axis we treated cells with the poly ADP-ribose polymerase inhibitor talazoparib and the alkylating agent temozolomide and detected synergistic activation of apoptosis only in the presence of mutant p53. Furthermore when minichromosome maintenance 2–7 activity was inhibited the synergistic activation of apoptosis was blocked. This mutant p53- poly ADP-ribose polymerase -minichromosome maintenance axis may be useful for theranostics.

## Personalized medicine: Mutated tumors respond to therapy

Mutations in the p53 tumor suppressor gene could offer a predictive biomarker of response to certain drugs in triple-negative breast cancer. Jill Bargonetti from Hunter College in New York, USA, and colleagues showed that mutant p53, which is expressed in more than 80% of patients with triple-negative breast cancer, interacts with and regulates hundreds of proteins, including those found in a complex needed for DNA replication. Members of this complex, called the minichromosome maintenance protein complex, interact with mutant p53—but less with wild-type p53. Bargonetti’s team targeted this pathway in mutated breast cancer cells with the PARP inhibitor talazoparib and the chemotherapeutic agent temozolomide. They observed synergistic cell killing with the two drugs, but only when the minichromosome maintenance protein complex was working and when p53 was mutated. These findings point toward a new strategy for personalizing therapy.

## Introduction

Missense mutations in the *TP53* gene often results in mutant p53 (mtp53) protein with gain-of-function (GOF) properties that are associated with multiple types of cancers, including lung and breast cancer.^1^ Mutations in p53 are found in 80% of triple negative breast cancers (TNBC).^2–4^ A number of studies have been carried out to elucidate the mtp53-associated breast cancer transcriptome but the mtp53-targeted proteome is less well studied.^5–8^ Mtp53 has not been found to interact with DNA site-specifically but has been found to interact with cancer cell DNA in association with other cofactors. Importantly mtp53 modifies chromatin structure to up-regulate vascular endothelial growth factor receptor 2^9,10^ and GOF mtp53 modifies major chromatin pathways by upregulating methyltransferase chromatin regulatory genes MLL1, MLL2, and the acetyltransferase MOZ.^11,12^ While changes in the transcriptome are a part of the mechanism of action of GOF mtp53, there are also transcription-independent mtp53 functions on chromatin that require further elucidation.

**Fig. 1:**
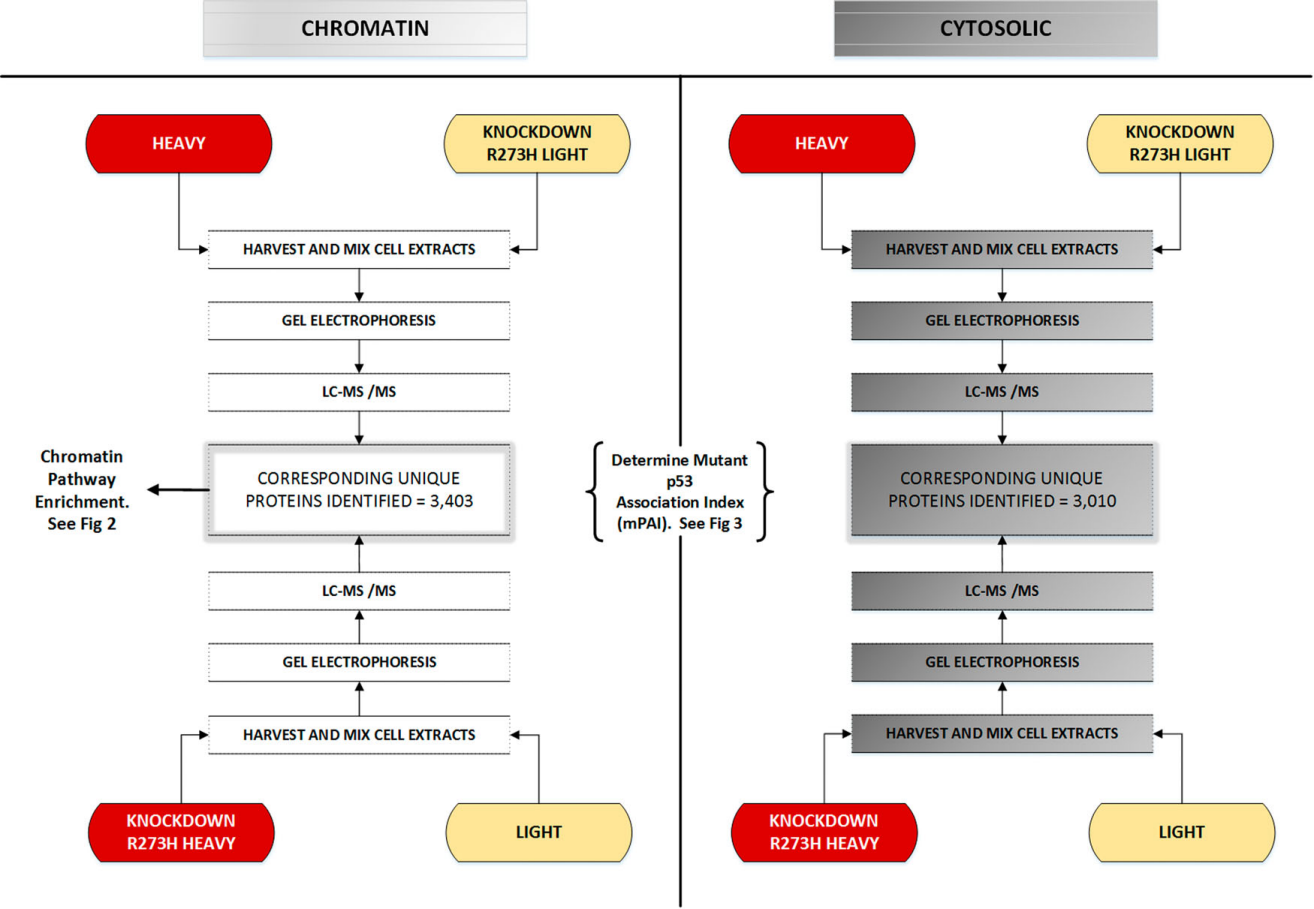
SILAC work flow for proteomic targets. Four independent LC-MS/MS experiments were carried out to compare the proteomes of chromatin and cytosolic proteomes with mtp53 knockdown. The work flow diagram briefly details the scientific steps from cell culture conditions to the identification of unique proteins. See Figs. 2 and 3 for identification of chromatin pathway enrichment and the mutant p53 association index for specific proteins and pathways compared for chromatin and cytosolic fractions

Very few studies have focused on the mtp53-associated proteome but new work strongly indicates that alternative experimental approaches are required to understand the complexity of the mtp53 pathway.^7,13^ A multiomics approach recently identified the proteasome machinery as a common target of missense mtp53.^7^ We are the only group to report on the influence of endogenous GOF mtp53 on the spatial segregation of the cancer cell proteome.^6^ The mtp53-associated cytosolic proteome targets include up-regulation of cytoplasmic poly ADP-ribose polymerase (PARP) when mtp53 is depleted^6^ and a decrease in the cytosolic mavelonate pathway enzymes (which is in agreement with previous transcriptome data).^5^ During validation of the spatially segregated proteins we discovered that down-regulation of mtp53 caused a chromatin-segregated decrease of PARP.^6^ We now report on the chromatin-segregated stable isotope in cell culture (SILAC) screen to identify the spatially restricted mtp53-targeted proteome of chromatin. We used a bioinformatics approach to compare the cytoplasmic and chromatin data sets (see Fig. 1 for the work flow). Recent work suggests that a key regulatory role for mtp53 on chromatin is to regulate transcription by chromatin remodeling,^12^ but we hypothesized that DNA repair and DNA replication could also be critical targets.

To our knowledge, there has been no direct evidence of GOF mtp53 regulating chromatin-mediated DNA replication and repair. Herein, we identified a mtp53-PARP-MCM chromatin axis by an unbiased bioinformatics screen of spatially segregated cytoplasmic vs. chromatin SILAC data from R273H mtp53 knockdown in TNBC cells. The enzyme PARP1 catalyzes the transfer of ADP-ribose to target proteins and plays a role in many cellular processes including transcription, DNA replication, and DNA repair.^14,15^ Herein, we validate the mtp53-PARP-MCM axis and found that blocking PARP1 may be an excellent therapeutic target for certain mtp53-expressing TNBCs.

## Results

### Gain-of-function mtp53 influences 3403 chromatin proteins

Stable isotope labeling in cell culture (SILAC) of the MDA-MB-468.shp53 cell line was carried out and mtp53 R273H was depleted by inducible shRNA expression in two independent reciprocal experiments. A work flow diagram (Fig. 1) shows the experimental approach that included cell fractionation and LC-MS/MS of heavy and light extract mixed at a 1:1 protein concentration ratio. For one experiment the mtp53 was depleted in the heavy label conditions (^13^C_6_ l-Lysine-2HCl and ^13^C_6_^15^N_4_ l-Arginine-HCl) and for the other mtp53 was depleted in the light label conditions. Chromatin fractionation was adapted from the Mendez and Stillman protocol.^6,16^ Following gel electrophoresis we used liquid chromatography and tandem mass spectrometry (LC-MS/MS) to identify the mtp53 protein targets associated with the chromatin fraction. We sequenced over 70,000 chromatin-associated peptides and compared the heavy/light ratio resulting from the depletion of mtp53 to determine how R273H knockdown reciprocally influenced the 3403 representative proteins. The chromatin mtp53 SILAC data were examined for gene set enrichment and then compared to the cytosol mtp53 targets determined in our previously published results.^6^ We carried out a bioinformatics comparison of the influence of mtp53 depletion on proteins in the cytosol to those affected on the chromatin.

### Gene set enrichment analysis indicates that the hexomeric pre-replicative MCM2–7 complex is the most highly enriched mtp53-associated chromatin complex

**Fig. 2:**
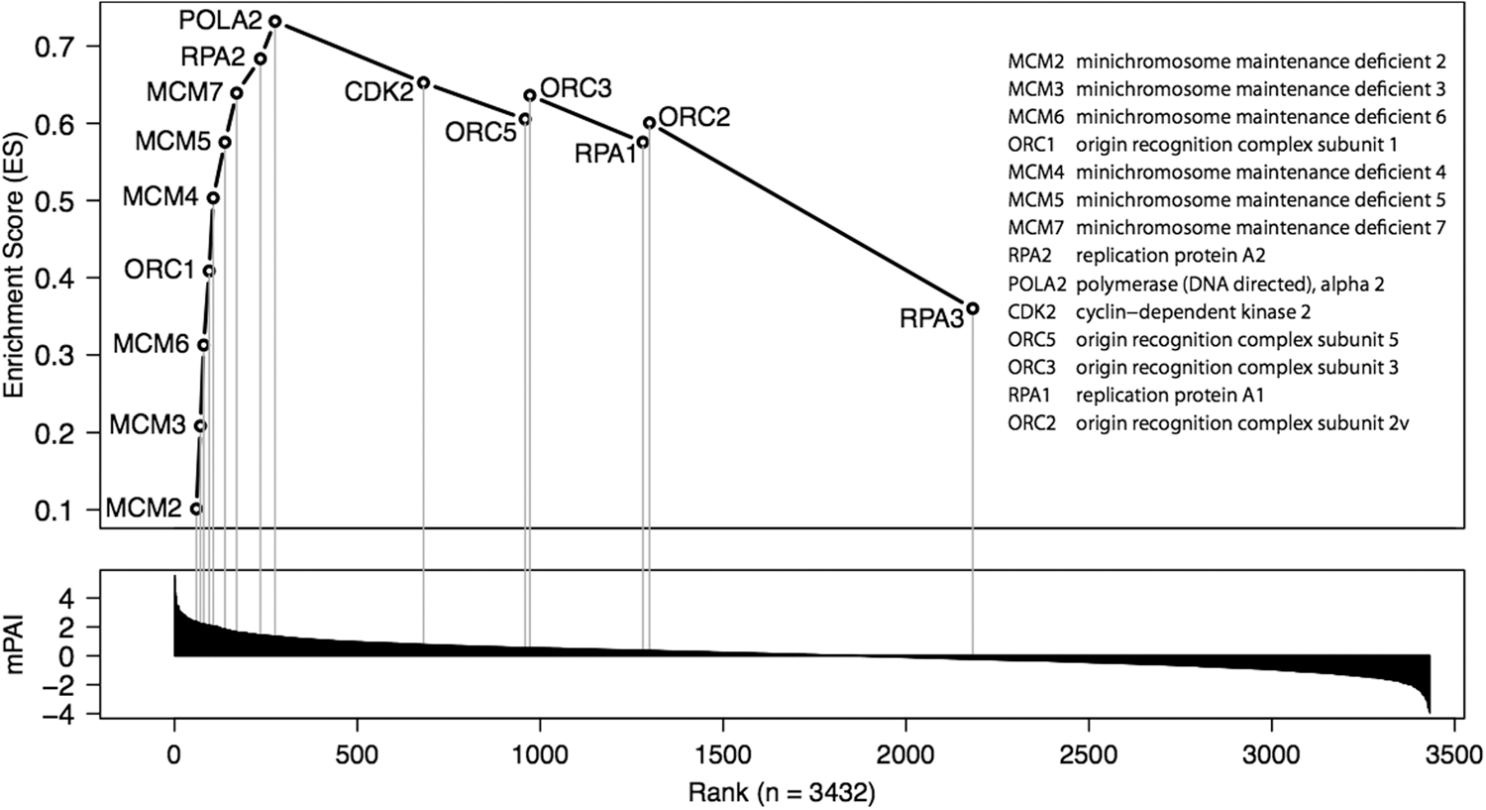
Knockdown of mtp53 identifies enrichment of the pre-replicative complex pathway. GSEA analysis revealed a total of 27 and 17 Reactome pathways that are positively and negatively, respectively, associated with mtp53 abundance at a nominal *P* value < 1%. Enrichment of five DNA replication-related gene sets and pathways including the pre-replicative complex consisting of 15 genes, shown here with their enrichment scores (*upper panel*) and mutant p53 association index (mPAI) ranks (*lower panel*). Full GSEA results are available at http://diverge.hunter.cuny.edu/~weigang/silac-chromatin-gsea/ (chromatin) and http://diverge.hunter.cuny.edu/Polotskaia_etal_2014/supp-table-s1/ (cytosol)

The gene set enrichment analysis was performed using the GSEA software (GSEA; version 2.0.14)^17^ to determine how chromatin associated proteins were influenced by mtp53. A defined set of genes associated with the proteins that showed concordant differences between the biological states of the mtp53 present, vs. the mtp53 absent, was determined with pathways defined by the Reactome Pathway Database (version 4.0).^18^ The GSEA analysis of the chromatin fraction revealed a total of 27 Reactome pathways that were positively associated with mtp53 abundance at a *P* value < 1%. Interestingly, a key pathway was the pre-replicative complex, chromatin enriched proteome pathway, which is a novel finding for mtp53 GOFassociations. The proteins in this pathway are (in rank order): MCM2, MCM3, MCM6, ORC1, MCM4, MCM5, MCM7, RPA2 and POLA2 (Fig. 2). The GSEA proteomic chromatin enriched pathway sets are shown in their entirety at the link: http://diverge.hunter.cuny.edu/~weigang/silac-chromatin-gsea/. The first three positive GSEA pathways in the list corresponded with electron transport, which did not directly correlate with a chromatin-associated pathway; we hypothesize this resulted from insoluble cellular components that were associated with the chromatin pellet. The fourth GSEA pathway associated with generic transcription pathways, which are currently the focus of many mtp53 GOF studies.^12^ However the fifth pathway identified a very specific chromatin associated pre-replicative pathway that has not yet been studied for mtp53 involvement. The GSEA analysis also demonstrated 17 Reactome pathways that were negatively associated with mtp53 abundance at a *P* value < 1%. By clicking on enrichment results in html format you will be directed to the positive association protein sets and negative association protein sets (Fig. 2). We are providing open access to this powerful data set.

### Distributions of mtp53 associated changes in the cytosol and on the chromatin indicate that the hexomeric MCM2–7 complex proteins reside in the double positive quadrant

**Fig. 3:**
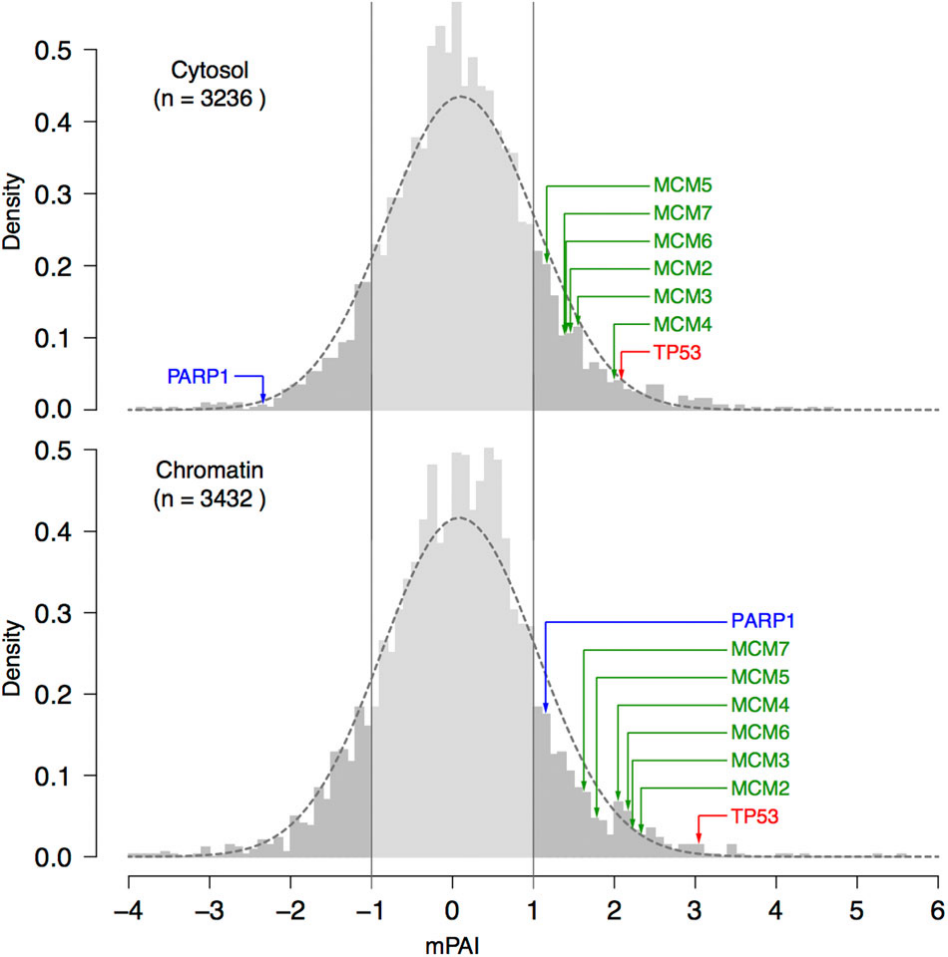
Distribution of mutant p53 association index (mPAI) scores in the cytosol (*top*) and chromatin (*bottom*) fractionations. Histograms of mPAI values (for equation see statistics in the methods) show close fit to normal curves (in *dashed lines*) obtained with the same mean (*µ* = 0.0060 for cytosol, *µ*= 0.085 for chromatin) and standard deviation (σ = 0.92 for cytosol, σ = 0.96 for chromatin). As expected (and as a negative control of the experiments and the mPAI statistic), most proteins are not significantly impacted by mtp53 depletion, showing −1 < mPAI < 1 (shaded in *light gray*). As a positive control, the mTP53 protein level (in *red*) shows significantly high positive mPAI values. As another positive control, the PARP1 levels (in *blue*) show contrasting mPAI values between the two fractionations, consistent with previous experimental results.^6^ The six components of the MCM2–7 complex (in *green*) show significant positive association with mtp53 in both cytosol and nucleus

The chromatin SILAC data were then compared to our previously published cytosol set http://diverge.hunter.cuny.edu/Polotskaia_etal_2014/supp-table-s1/. In order to further summarize and quantify the degree of under- and over-expression of proteins from the reciprocal knockdown experiments in the different sub-cellular fractions of the breast cancer cells we defined a mtp53 association index (mPAI: see “Methods” for the statistical analysis). Values of mPAI obtained from both the cytosol and chromatin fractionations were normally distributed with a mean close to zero and a standard deviation close to one, conforming to the expectation that abundance of the majority of proteins were indeed not impacted by mtp53 knockdown (Fig. 3). We thereby identified proteins with mPAI > 1.0 as those displaying significant positive association with mtp53 abundance and those with mPAI < −1.0 as showing significant negative association with mtp53 abundance. This was in agreement with the fact that mtp53 knockdown did not influence the level of the majority of the proteins in either the cytosol or chromatin sub-cellular fractions. Moreover, in both sub-cellular fractions the standard deviation of the mPAI was close to one and mtp53 itself showed an mPAI value of greater than 2.0 (*z*-score > 2.0). The mtp53 mPAI index was 3.0 on the chromatin, which was higher than the positive 2.1 value identified in the cytosol. The mtp53 mPAI served as excellent internal positive control as its levels necessarily were reduced by shRNA mediated knockdown. Poly ADP ribose polymerase (PARP) was associated with the chromatin when mtp53 levels were high and redistributed to the cytosol when the mtp53 was low as expected and the mPAI for PARP reflected this as a negative value for the cytosol and a positive number for the chromatin (Fig. 3). In support of our previous data, we determined that PARP had a positive mPAI on the chromatin of 1.2 and a negative mPAI in the cytosol of −2.3. Therefore in addition to providing a new powerful data set we have identified a potentially important mtp53 protein pathway that is involved in regulation of DNA replication.

### Comparison of the nuclear and cytosol proteomes displays a double positive mtp53 influence on the MCM 2-7 hexomeric complex

**Fig. 4:**
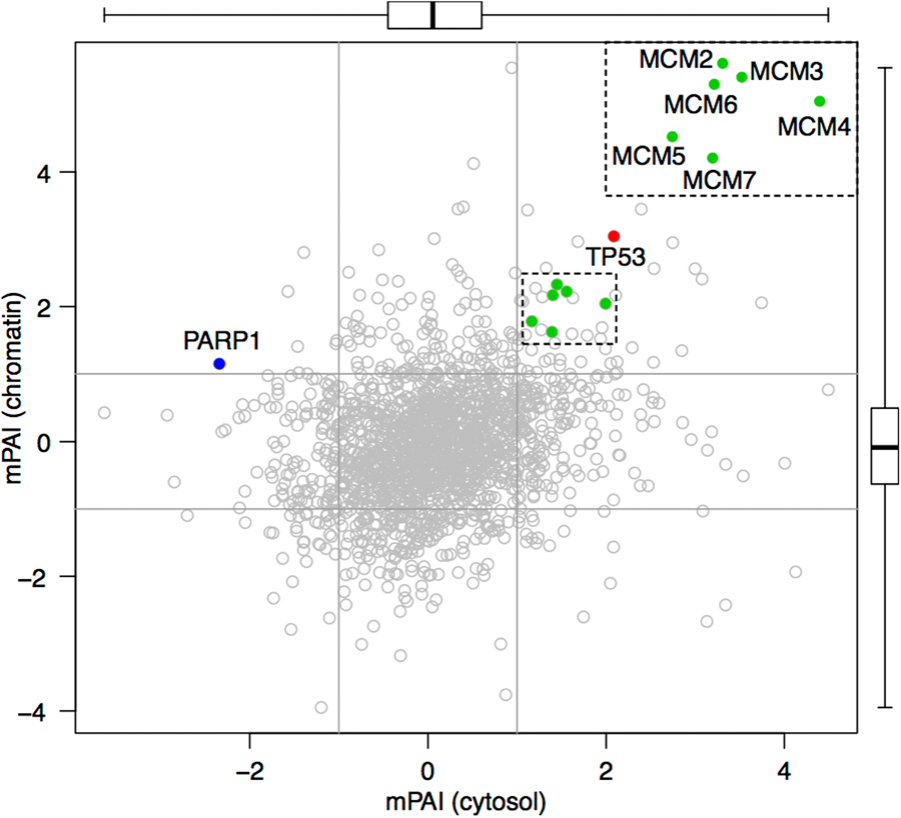
A double-positive mtp53 association seen for all MCM2-7 complex proteins in cytosol (*x*-axis) and nucleus (*y*-axis). Each dot (*n* = 1778) represents a protein with its position determined by its mPAI values in the cytosolic (*x*-axis) and the chromatin fractionations (*y*-axis). mPAI values were averaged if multiple peptides of the same protein were identified. Two side boxplots show the median, the *lower* and *upper* quartiles, and the range of mPAI values. The majority of points fall inside the *x* = −1, *x* = 1, *y* = −1, and *y* = 1 lines, indicating that abundance of most proteins are not significantly impacted by mtp53 knockdown in either fractionation. The mtp53 and the six members of the MCM2–7 complex fall in the *top right* quadrant, where protein levels are positively associated with mtp53 levels in both fractionations. PARP1 falls in the *top left* quadrant showing negative association with mtp53 in the cytosol but positive association in the nucleus, consistent with our previous experiment result.^6^ A searchable, zoomable, and clickable scatter plot of mPAI values for 4798 genes and 1330 associated pathways and gene sets is available at http://diverge.hunter.cuny.edu/~weigang/mpai-browser/

The mPAI for the entire mtp53-influenced proteome in the cytosol vs. the mtp53-influenced proteome on the chromatin were graphed as coordinates of the chromatin proteome on the Y-axis and the cytosol proteome on the X-axis. This resulted in a scatter plot with four quadrants demonstrating differentially influenced mtp53 associated proteins. Figure 4 shows a representative image with all the dots as grey shades, p53 as a prominent red dot, the MCM2–7 helicase subunits as green dots (zoomed in in upper right), and PARP as a blue dot. An interactive searchable scatter plot is part of the Supplementary Data http://diverge.hunter.cuny.edu/~weigang/mpai-browser/. Each dot represents a protein and its mPAI in the cytosol and chromatin. The four p53-influenced quadrants are (a) double positive in the top right, (b) chromatin positive and cytosol negative in the top left, (c) double negative in the bottom left, and (d) cytosol positive with chromatin negative in the bottom right. The mtp53 protein is by definition a double positive signal and it is highlighted as a red dot (TP53, Fig. 4). The MCM2–7 pre-replication complex proteins are shown in green and were all situated as double positives (Fig. 4). The PARP1 protein appeared in the upper left quadrant and is highlighted as a blue dot. PARP1 showed a negative association with mtp53 in the cytosol and a positive association on the chromatin, consistent with our previous experimental results. The center of the scatter plot corresponds to proteins that are unchanged by p53 knockdown. While the majority of proteins are unchanged by the knockdown of mtp53 key proteins and pathways including those involved in DNA replication and repair are strongly implicated.

### Mutant p53 interacts with members of the MCM hexomeric complex on chromatin

**Fig. 5:**
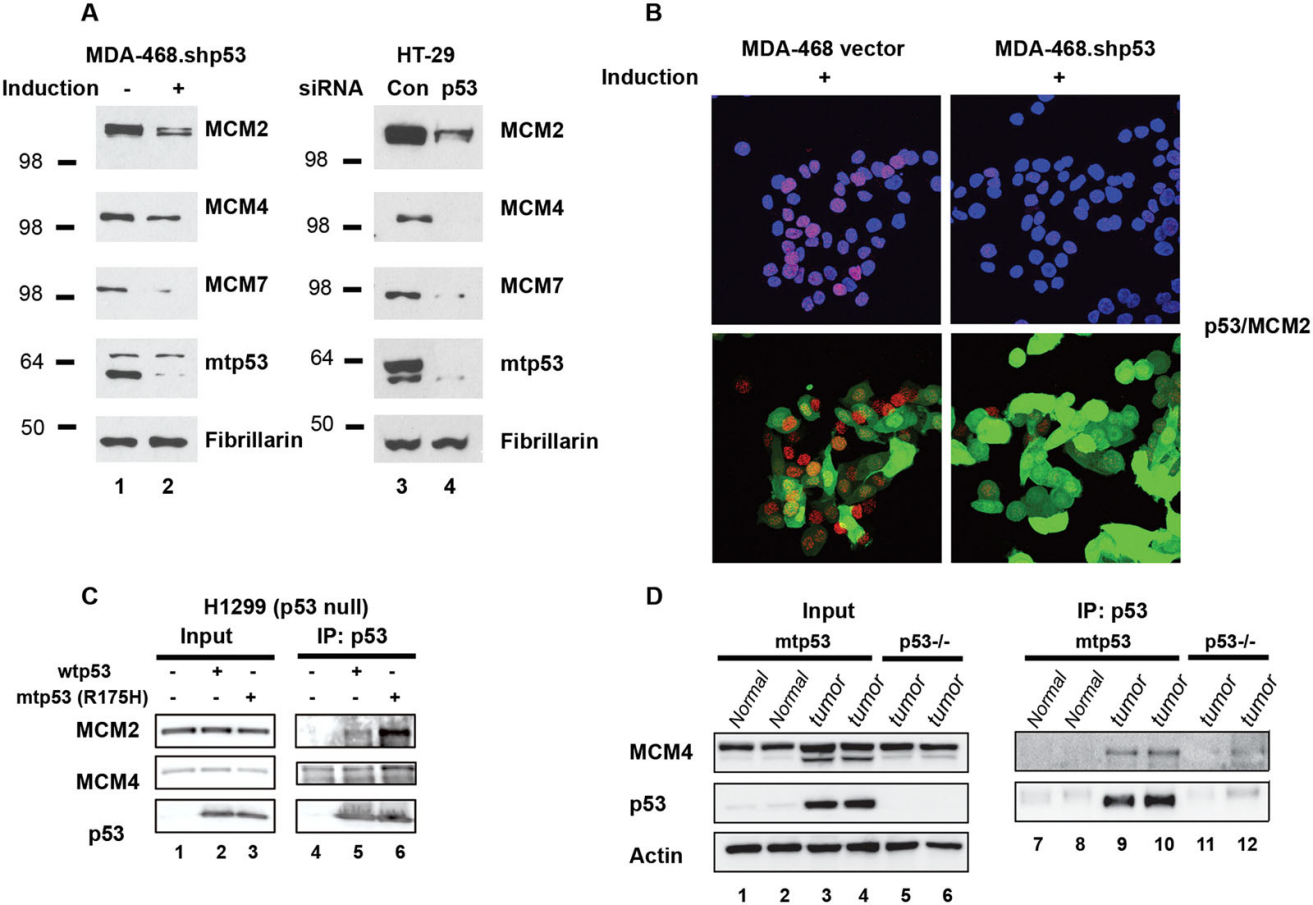
Mutant p53 associates with MCM2, MCM4 and MCM7 on chromatin. **a** Protein levels of MCM2, MCM4, MCM7, mtp53 and fibrillarin in the chromatin fraction were determined by Western blot analysis in MDA-468.shp53 cells grown in the presence or absence of 8 μg/ml of doxycycline for 12 days, and HT-29 colon cancer cells transfected with p53-siRNA (p53) or control siRNA (Con). **b** Analysis of p53/MCM2 complexes (*red*) by immunofluorescence microscopy in combination with in situ proximity ligation assay (PLA) in MDA-468 vector and MDA-468.shp53 cells grown in the presence of 8 μg/ml of doxycycline for 12 days. DNA was counterstained with DAPI (*blue*) and GFP (*green*) was an indicator of doxycyline-mediated induction. The z stack confocal maximum intensity projection images of p53/MCM2 and DAPI, p53/MCM2 and GFP are shown. **c** Co-immunoprecipitation (co-IP) of MCM2 and MCM4 with exogenously expressed mtp53 (R175H) and wtp53 in H1299 cells. Whole cell lystates of H1299 cells transfected with wtp53 or mtp53 were incubated with anti-p53 antibody followed by immunoblot with anti-MCM2 or anti-MCM4 antibodies. **d** Co-IP of MCM4 and mtp53 in thymic lymphomas from mtp53 (Trp53^R172H/R172H^) mice. Thymic lymphomas from mtp53 mice and p53−/− mice as well as normal thymic tissue from mtp53 mice were subjected to co-IP assays using anti-p53 antibody followed by immunoblot with anti-MCM4 antibody

To verify that the R273H mtp53 levels influenced multiple MCM hexomeric proteins on chromatin in different cells, we reduced GOF mtp53 levels in MDA-MB-468 cells and HT-29 cells and used Western blot analysis to examine MCM2, MCM4, and MCM7 (Fig. 5). When mtp53 was decreased the chromatin-associated levels of MCM2, MCM4, and MCM7 were also decreased (Fig. 5a). To further examine this interaction in situ and to determine if the mtp53 was co-localized with the MCM2–7 we used proximity ligation assay (PLA) with confocal microscopy detection.^19^ To our knowledge we are the first group to use antibodies in PLA to detect the interaction of mtp53 and MCM2. Strong nuclear co-localization of mtp53 and MCM2 was apparent and this signal was drastically reduced by the knockdown of mtp53 (Fig. 5b). The results from the PLA documented an interaction between mtp53 and MCM2 that was restricted to the subcellular nuclear zone. Our data showed that mtp53 R273H interacted with MCM2 in the nucleus and made us interested in seeing if the interaction of missense mtp53 with MCM2 was a more general phenomenon. In order to address the interaction of other mtp53 isoforms and wild-type p53 with MCM2 we compared the MDA-MB-468 PLA signal to those seen in a number of other cell lines (Supplemental Figure 1). The confocal microscope settings were kept constant in order to have a direct comparison. We observed a strong PLA signal between R280K mtp53 and MCM2 in MDA-MB-231 cells and this was reduced by mtp53 knockdown. We also observed a strong PLA signal between R248Q mtp53 and MCM2 in HCC70 cells, which again was reduced by mtp53 knockdown. Interestingly, we detected some MCM2 interacting with the low level wtp53 in MCF-7 cells and this reaction was stable. Therefore the high concentration of different missense mtp53 on the chromatin in cancer cells corresponds to a strong PLA signal with MCM2, and even low-level wtp53 can be found in close proximity to MCM2. A previously published immunoprecipitation screen of mtp53 R175H found an interaction with MCM proteins that was reported only in the Supplementary Data section.^20^ We found that exogenously expressed mtp53 R175H, and to a much lesser extent wild-type p53, interacted with both MCM2 and MCM4 (Fig. 5c). Mice with the analogous human R175H knockin mutation (Trp53^*R172H/R172H*^) develop lymphomas.^21,22^ We also found that the mtp53 in these mouse tumors interacted with MCM4 (Fig. 5d). In mice with mtp53 R172H, the protein is low in normal tissue and is only found stable and highly expressed in tumor tissue.^23^ Therefore it is not surprising that there was very little mtp53 evident in the input, or immunoprecipitation samples from normal tissue (Fig. 5, lanes 1, 2, 7, and 8). The fact that we observed a stronger interaction between mtp53 R175H and MCM2, and a weaker interaction between wtp53 and MCM2, corresponds with our observations for comparative PLA analysis for multiple breast cancer cell lines (Supplementary Figure 1). The MCM4 interaction in the co-immunoprecipitation was more difficult to assess due to poor antibody specificity, but nevertheless looked strongest for mtp53. From these data we conclude that all forms of p53 can be found in close proximity to MCM proteins but that a higher level of oncogenic mtp53 in cancer cells corresponds to a much more robust signal for the proximity interaction with MCM proteins. The fact that we do not see strong enrichment for co-immunoprecipitation of MCM proteins, but see a strong proximity interaction suggests that the mtp53–MCM interaction is not due to a strong direct protein–protein interaction.

### Activation of apoptosis and PARP trapping is mitigated by knockdown of mtp53 or inhibition of MCM2–7

**Fig. 6:**
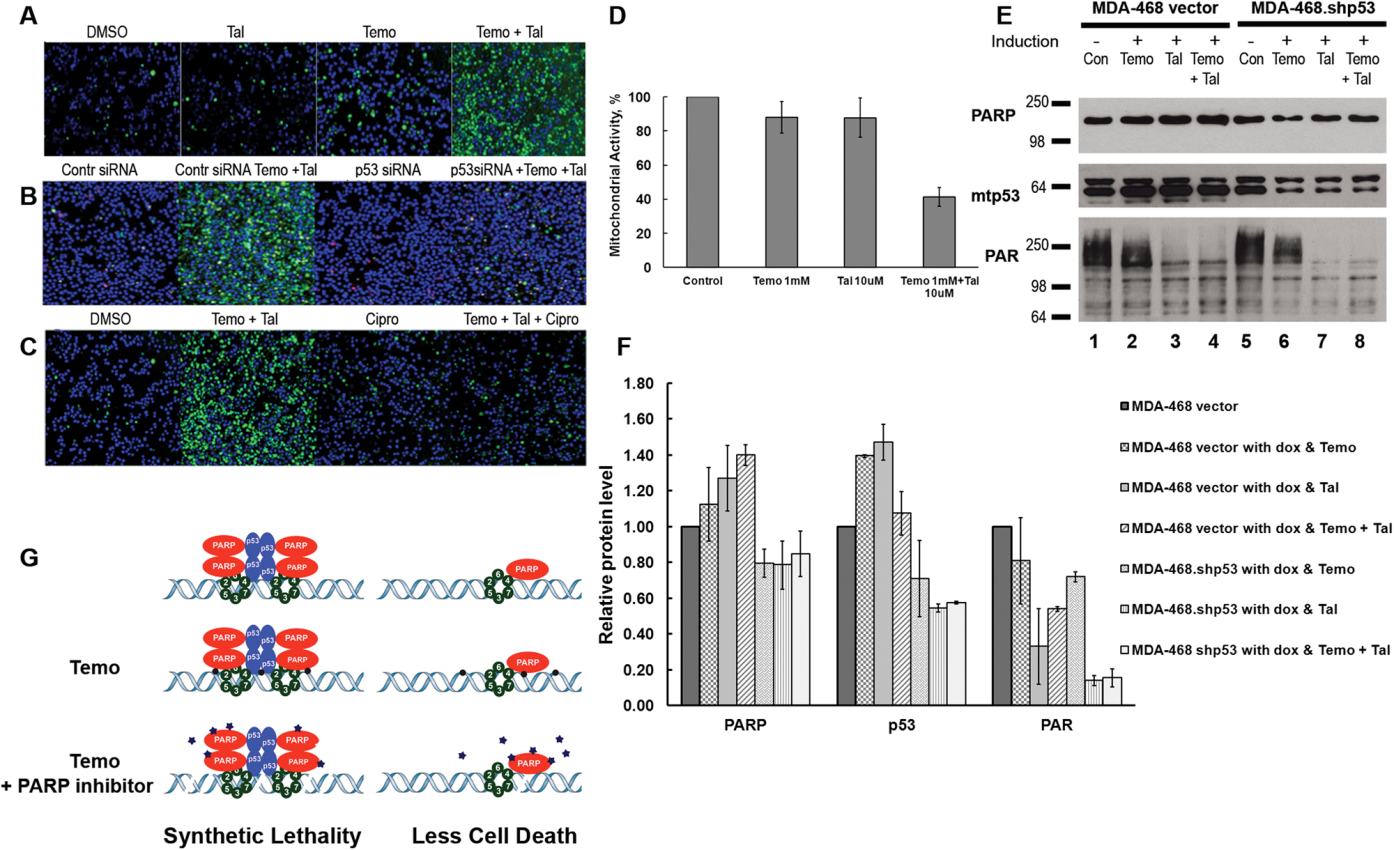
Activation of apoptosis and PARP trapping is mitigated by knockdown of mtp53 or inhibition of MCM2–7. Confocal maximum projection of live-cell imaging in MDA-468 cells (**a**, **c**) or MDA-468 cells transfected with p53-siRNA or control siRNA (**b**) treated for 24 h with temozolomide (Temo), talazoparib (Tal), combination (Temo + Tal) or ciprofloxacin (Cipro). Apoptotic cells (*green*) were detected by activated caspase 3/7 *green* detection reagent and DNA was counterstained with DAPI (*blue*). *Red* fluorescence was Propidium iodide staining. **d** MTT assay shows reduction of mitochondrial activity after combination treatment of Temo plus Tal. **e**, **f** PARP trapping and PARylation with and without mtp53 after 4 h treatment with Temo, Tal or combination (Temo + Tal). Protein levels of PARP, mtp53 and PARylated proteins in the chromatin fraction were determined by Western blot analysis in MDA-468 vector and MDA-468.shp53 cells grown in the presence or absence of 8 μg/ml of doxycycline for 12 days. The Western blot is a representative image. The histogram is based on signal intensity (quatified using Image J software) from two independent experiments and normalized to untreated cells set as one. **g** Schematic model of the mtp53 dependent synthetic lethality by the combination of talazoparib plus temozolomide

We previously saw that the inhibition of PARP was more cytotoxic in the presence of mtp53 than in its absence.^6^ We hypothesized that this might be due to a mtp53–PARP–MCM interaction at damaged DNA. Synergistic activity is seen when the PARP inhibitor talazoparib is used in combination with the DNA damaging agent temozolomide in BRCA1 mutant cells.^24^ It has been shown that wtp53 expression decreases sensitivity of breast cancer cells to PARP inhibition^25^ and ciprofloxacin blocks the MCM2–7 complex.^26^ We asked if increased cytotoxity of PARP inhibition could be detected in the presence of mtp53 if DNA was damaged by alkylation. We predicted that there would be synergistic activation of apoptosis of the breast cancer cell lines with mtp53 in the presence of talazoparib plus temozolomide because this alkylating agent has been shown to provoke PARP trapping.^27^ We found that combination treatment with talazoparib plus temozolomide induced synergistic activation of apoptosis only in the presence of mtp53 and only when MCM2–7 processivity was not inhibited by ciprofloxacin (Fig. 6a–c). This was detected by live cell confocal microscopy scoring for activated caspases 3 and 7 (Fig. 6a–c). PARP inhibition by talazoparib plus DNA damage with temozolomide resulted in synergistic cell killing of MDA-MB-468 breast cancer cells that are wild-type BRCA1 and R273H mtp53 (Fig. 6a; apoptotic cells are green stained with active caspase 3 and 7). Moreover, when R273H mtp53 expression was depleted by siRNA, or MCM2-7 was inhibited by ciprofloxacin this synergistic activation was blocked (Fig. 6b and 6c). In addition, cell viability reduced 59% in talazoparib plus temozolomide treatment compared to non-treated cells (Fig. 6d). We also found, as predicted, that combination treatment with talazoparib plus temozolomide increased PARP trapping on the chromatin and this was mitigated by the knockdown of mtp53 (Fig. 6e and 6f). Moreover, depletion of mtp53 reduced the poly-ADP-ribosylated (PAR) proteins in the combination treatment with talazoparib plus temozolomide (Fig. 6e). Therefore mtp53 R273H and processive MCM2–7 are required for the higher than additive killing seen when cells are treated with talazoparib plus temozolomide.

## Discussion

High levels of mtp53 are found in over 50% of all human tumors from patient samples.^1^ Somatic mutations in only three genes occur at greater than 10% incidence across all different subtypes of breast cancers, and one of these is mutation in the *TP53* gene.^2^ More than 80% of TNBCs contain mtp53 protein.^2–4^ As far back as 1984 it was reported that the “oncogene” p53 cooperated with *ras* to transform cells;^28,29^ however, we still do not use mtp53 as a diagnostic or treatment-mediated paradigm. TNBCs may serve as an ideal paradigm for this approach. There are a number of high-occurrence “hot spot” mutations found in the *TP53* gene that result in amino acid substitutions that inhibit the site-specific DNA binding activity of p53.^8^ Some *TP53* mutations contribute to breast cancer metastasis because of loss of p53 tumor suppressor activity, many missense *TP53* mutations cause new-found GOF oncogenic activities that range from transcriptional activation of target genes that promote tumorigenesis, to the inhibition of p53 family members p63 and p73.^30^ The GOF mtp53 proteins have a prolonged half-life and are highly expressed in cancer cells.^1,23^ While mtp53-mediated regulation is known to occur in part by activation and repression of gene transcription,^8,30–32^ mounting evidence including data from our lab indicates that other biochemical functions exist for mtp53.^6,13,31^ Improved detection of proteomic signal transduction changes are observed with sub-cellular fractionation experiments of SILAC followed by LC-MS/MS.^33,34^ We are the only group to study mtp53-proteome interactions in the context of sub-cellular architecture, which is critical for monitoring stability of proteins based on location. To carefully analyze the proteomic data we designed an algorithm to assay four data sets generated by inducible mtp53 knockdown in conjunction with SILAC and mass spectrometry to rate the mtp53 association index (mPAI). The mPAI points were graphed in an interactive four-quadrant map of proteomic changes to compare cytosol and chromatin targets http://diverge.hunter.cuny.edu/~weigang/mpai-browser/. The genes and pathways associated with mtp53 were identified using GSEA and can be searched with this interactive tool online. When MCM is typed in the gene search tool you will see that all six members of the hexomeric complex are found in the double positive quadrant. When the word “replicative” is typed into the search pathway function the pre-replicative pathway will appear and when the point is clicked all six of the MCM proteins will show up again. The strength of this online analysis tool is that, this is the first time many other mPAI pathways are presented and they are yet to be validated. Herein, we validate the mtp53–PARP–MCM pathway. We identified that mtp53 depletion also depleted chromatin-associated PARP and all members of the MCM2-7 hexomeric complex. We are the first to show that mtp53 influenced MCM chromatin levels in multiple cancer cell lines and directly associated with MCM2 in nuclei as seen by confocal microscopy PLA (Fig. 5).

TNBCs are resistant to a number of different treatments; temozolomide is one of the chemotherapeutic drugs TNBCs are resistant to.^35^ It is clear that properties in addition to BRCA1/2 status dictate the sensitivity to PARP inhibitors^36^ and we hypothesize that mtp53 status (with specific hot spot mutations) is a critical determinant. Synergistic activity is seen with the PARP inhibitor talazoparib in combination with the DNA modifier temozolomide.^24^ PARP is recruited to DNA damage sites in chromatin to block transcription and facilitate DNA repair^34^ and recently MCM2-7 was also found to participate in DNA repair.^37^ Our study is the first to directly show that the synergistic activity of temozolomide plus talazoparib is dependent on the expression of mtp53 and the processivity of MCM2–7 (Fig. 6). Interestingly the proteomic study finding BAG2 stabilizes mtp53 also identified MCM proteins interacting with mtp53, and the proteomic study finding MCM2–7 is involved in DNA repair also found BAG2 interacting with the complex.^20,37^ This suggests that stable mtp53 may help recruit MCM2–7 and PARP proteins to chromatin in order to help cancer cells survive during replication stress. While PARP inhibitors have been used to target breast cancers with BRCA1 mutations,^38^ they have not been approved for use in cancers that have mutation in the *TP53* gene. Breast cancers with BRCA1 mutations include many TNBCs, however PARP inhibitors have not shown a direct correlation of effectiveness directly related to BRCA1 and BRCA2 mutations in TNBCs.^36^ MDA-MB-468 cells and HCC70 cells do not have BRCA mutations and they are more sensitive to PARP inhibitors than some breast cancer cell lines that have BRCA mutations. Importantly both of these TNBC cell lines express high levels of mtp53, however a correlation between mtp53 status and PARP activity before now had not been determined. Recent work has shown that the cytotoxicity of PARP inhibitors requires that the inhibitors trap the PARP enzyme onto the chromatin.^27,39^ Importantly, we found that in the presence of mtp53, but not in its absence, combination treatment with the PARP inhibitor talazoparib plus the DNA damaging agent temozolomide resulted in efficient PARP trapping and apoptosis induction (Fig. 6).

It is of interest and important to determine if the temozolomide plus talazoparib combination strategy works in vivo with a specificity for tumor cells possessing specific p53 missense mutations. We found that while mtp53 is highly associated with MCM2 on the chromatin (Fig. 5b and Supplementary Figure 1A), the mutation R273H associated with the highest level of synergistic killing by the combination treatment, and the sensitivity for cells with other p53 missense mutations varied (Supplementary Figure 1B). There have been demonstrations of remarkably synergistic activity of the PARP inhibition by talazoparib plus temozolomide in a subset of pediatric Ewing Sarcoma xenografts.^24^ The genomic landscape of Ewing Sarcoma shows an aggressive subtype with TP53 mutations.^40^ The p53 status has been reported for many of the cell lines used for the Ewing Sarcoma xenograft models, and there is not a direct correlation between the p53 mutation status and those cell lines that are sensitive to combination treatment vs. those that are not.^24^ Cancer cell sensitivity to combination treatment may be specific for certain p53 missense mutations in collaboration with other driver or passenger mutations. We previously documented that the depletion of mtp53 in MDA-MB-231 cells does not reduce MCM protein on the chromatin.^6^ Herein, we saw that there was an interaction between mt53 R280K and MCM2 in MDA-MB-231 cells; however combination temozolomide plus talazoparib treatment of MDA-MB-231 cells did not cause synergistic killing (Supplementary Figure 1B). It is possible that missense mtp53, in different contexts, influences PARP and MCM in different ways. Experiments are needed to elucidate the relationship between different p53 missense mutants, and accessory proteins, for influencing PARP and MCM2-7 structure and function.

Homologous-recombination-deficient tumors are dependent on DNA-replication repair mechanisms that are more sensitive to PARP inhibitors.^41^ It is possible the certain missense mutants of p53 block homologous-recombination in humans, as the p53 in *C. elegans* inhibits nonhomologous end joining while promoting high fidelity homologous recombination.^42^ Our results describe the close proximity between mtp53 and the replication initiator mini chromosome maintenance complex MCM2-7 on replicating DNA. However, they suggest that each missense mutation has to be evaluated for its specific activity. Researchers have found a way to reactivate mtp53 to become wild-type like, but this reactivation is allele specific for R175H.^43,44^ The ability to target a characteristic of multiple mtp53 proteins will enable using the newfound mtp53 activities to be used against tumorigenesis. Our results implicate an interaction of stable mtp53 at replication forks, and with PARP on the chromatin that can be used to sensitize cancer cells to die. We saw that the processivity of the MCM2-7 complex was required for synergistic mtp53-dependent induction of apoptosis by the combination of talazoparib plus temozolomide (see model in Fig. 6g). The MCM2–7 multi-subunit helicase participates in driving DNA replication and improves replication under stressful conditions. This may be the connection between mtp53 and MCM2–7 facilitating the synthetic lethal function of PARP inhibition in treating TNBC. The disruption of p53 by mutation often allows the subverted protein to interact with normal partners of wild-type p53 but differentially influences the outcome.^45^ It remains to be determined if PARP and MCM2–7 will be added to the list of proteins that are influenced by wild-type and mtp53 in opposing ways or if this is a new paradigm. Our findings demonstrate a connection between mtp53 expression in TNBC and the ability to target cells with the combination therapeutic drug protocol previously intended for BRCA1 mutated cancers. Taken together, our findings suggest that certain mtp53 missense mutations drive PARP trapping and then MCM2–7 helps to facilitate the increased cytotoxicity of PARP inhibitors plus temozolamide. This data also suggests that the treatment of TNBC, with specific mtp53 proteins, by PARP inhibitors plus temozolamide may have promising therapeutic effects and therefore the use of mtp53 status in TNBC may be a predictive marker for combination PARP-trapping therapy response.

## Methods

### Statistical analysis

We quantified the degree of under- and over-expression of proteins from the reciprocal knockdown experiments in the different sub-cellular fractions of the breast cancer cells by defining a mPAI. The mPAI was defined as

$${\rm{mPAI}}\,=\,{\mathrm{log}}_{2}\left[{\left(\frac{H}{L}\right)}_{{\rm{exp1}}}\right]-{\mathrm{log}}_{2}\left[{\left(\frac{H}{L}\right)}_{\exp 2}\right],$$

where ${\left(\frac{H}{L}\right)}_{\exp 1}$ is the ratio of peptide abundance in an experiment in which the control cells were labeled with the heavy isotope and the mtp53 knockdown cells were labeled with the light isotope, and ${\left(\frac{H}{L}\right)}_{\exp 2}$ is the corresponding ratio in the reciprocally labeled experiment. The use of logarithm with base two converts these ratios to the unit of fold changes between the control and the knockdown cells. When abundance of a protein is not affected by mtp53 knockdown, both *H*/*L* ratios are expected to be close to one, resulting in an mPAI ~ 0. For a protein with abundance increased by the presence of mtp53, ${(\frac{H}{L})}_{\exp 1}$is expected to be >1 and ${\left(\frac{H}{L}\right)}_{\exp 2}$ < 1, resulting in an mPAI > 0. Conversely, mPAI was expected to be <0 for a protein with abundance decreased by the presence of mtp53.

#### Reagents

Doxycyclin, aprotinin, leupeptin, DTT, temozolomide and ciprofloxacin were obtained from Sigma, Talazoparib BMN 673 from Selleckchem.

CellEvent Caspase-3/7 Green and ReadyProbes Cell Viability Imaging Kit Blue/Red for Live Cell Imaging were obtained from Life Technologies.

Duolink in situ red kit goat/rabbit (Sigma) was used for PLA assay.

#### Cell lines

MDA-MB-468, H1299 and HT-29 cell lines were obtained from ATCC and cultured in DMEM or McCoy’s (for HT-29) medium (Invitrogen), supplemented with 10% FBS (Gemini, West Sacramento, CA, USA) and 50 U/ml penicillin and 50 µg/ml streptomycin (Mediatech). Cell lines with the inducible p53 knockdown were generated and described previously.^5,46,47^ To induce shRNA expression cells were treated with 8 µg/ml of doxycyclin (Dox) for the time periods indicated in the figure legends, fresh medium with Dox was supplemented every 48 h.

#### Antibodies

Anti-human p53 mouse 1:1:1 mix of hybridoma supernatants pAb421, pAb240, and pAb1801 (N-terminus, Central and C-terminus regions respectively), rabbit anti-Actin (Sigma); mouse anti-Fibrillarin (AbCam), mouse anti-PARP-1 (Santa Cruz), rabbit anti-PAR (Millipore/Calbiochem), anti-MCM2, MCM4, MCM7 (Cell Signaling), secondary antibody: anti-mouse and anti-rabbit HRP-conjugated (Sigma).

#### Sub-cellular fractionation

Cells were harvested and fractionation was performed using the Stillman protocol.^16^ Briefly, cells were scraped from the plates, rinsed with cold PBS twice and pelleted by centrifugation in 50 ml tubes at 1000 rpm 5 min. Cell pellets were resuspended in buffer A (10 mM HEPES pH 7.9, 10 mM KCl, 1.5 mM MgCl_2_, 0.34 M sucrose, 10% glycerol, 1 mM DTT, 0.5 mM PMSF, 2 µg/ml leupeptin, 8.5 µg/ml aprotinin) with 0.1% Triton X-100. After 5 min incubation on ice cells were transferred to Eppendorf tubes and spun down at 3600 rpm for 5 min at 4 °C. The supernatant was spun down for an additional 5 min at 13,000 rpm at 4 °C to clarify (Cytoplasmic Fraction). Pellets were washed two times with Buffer A by centrifugation at 3600 rpm for 5 min at 4 °C. The nuclear pellet was resuspended in Buffer B (3 mM EDTA, 0.2 mM EGTA, 0.5 mM PMSF, 2 µg/ml leupeptin, 8.5 µg/ml aprotinin) and incubated on ice 30 min with vigorous vortexing every 5 min and spun down at 4000 rpm for 5 min at 4 °C. The supernatant was nuclear soluble proteins. The pellet enriched in chromatin, was washed two times with Buffer B, resuspended in buffer B and sonicated three times for 30 s followed by 30 s rest on ice (Chromatin Fraction). Samples were stored at −80 °C.

#### Gel electrophoresis and immunoblotting

Proteins were separated using 10% SDS-PAGE and transferred to a nitrocellulose membrane. The membrane was blocked in 5% non-fat milk solution in PBS/0.1% Tween 20 and probed overnight at 4 °C. Washes were done with PBS/0.1% Tween 20 solution. Secondary anti-mouse or anti-rabbit antibody (Sigma) was applied to the membrane for 1 h at room temperature and the membrane was washed three times. Protein signal was visualized by chemiluminescence using the Super Signal West Pico Kit (Pierce) and detected after exposure for autoradiography to Hyblot CL films (Denville Scientific).

### Quantitative proteomics by stable isotope labeling in cell culture SILAC mass spectrometry

For SILAC mass spectrometry, we used Protein Quantitation Kit—DMEM (Pierce) with ^13^C_6_ l-Lysine-2HCl and added to the media a second amino acid ^13^C_6_^15^N_4_ l-Arginine-HCl (Pierce) for double labeling. Cells were passaged for at least five cell doublings by splitting cells when required and isotope incorporation efficiency was determined by MS analysis. MDA-468.shp53 R273H depleted and non-depleted cells were cultured in media containing either non-labeled or labeled amino acids, harvested, fractionated, cytoplasmic, or chromatin fractions were mixed at 1:1 ratio, separated by SDS-polyacrylamide gel electrophoresis, stained with GelCode Blue Stain Reagent (Thermo Scientific), and 15 gel sections excised with in situ trypsin digestion of polypeptides in each gel slice performed as described.^48^ The tryptic peptides were desalted using a 2 µl bed volume of Poros 50 R2 (Applied Biosystems, CA) reversed-phase beads packed in Eppendorf gel-loading tips.^49^ The purified peptides were diluted to 0.1% formic acid and each gel section was analyzed separately by microcapillary liquid chromatography with tandem mass spectrometry using the NanoAcquity (Waters) with a 100-μm-inner-diameter × 10-cm-length C18 column (1.7 um BEH130, Waters) configured with a 180-µm × 2-cm trap column coupled to a Q-Exactive mass spectrometer (Thermo Fisher Scientific). Key parameters for the mass spectrometer were: AGC 3 E6, resolution 70,000. Tandem mass spectrometry fragmentation spectra were searched for protein identification using the Andromeda search engine (http://maxquant.org/) against the reversed and concatenated IPI_HUMAN protein database (v3.87). One unique peptide was required for high-confidence protein identifications and a minimum ratio count of two peptides (one unique and one razor) were required for SILAC ratio determination. Normalized SILAC ratios (H/L) were used for subsequent analysis. All MS/MS samples were analyzed using MaxQuant (Max Planck Institute of Biochemistry, Martinsried, Germany; version 1.3.0.3) at default settings with a few modifications. The default was used for first search tolerance and main search tolerance: 20 and 6 ppm, respectively. Labels were set to Arg10 and Lys6. MaxQuant was set up to search the reference human proteome database downloaded from Uniprot on April 2, 2013. Maxquant performed the search assuming trypsin digestion with up to two missed cleavages. Peptide, Site, and Protein FDR were all set to 1% with a minimum of 1 peptide needed for identification but two peptides needed to calculate a protein level ratio. The following modifications were used as variable modifications for identifications and included for protein quantification: Oxidation of methionine, acetylation of the protein *N*-terminus, phosphorylation of serine, threonine and tyrosine residues, and propionamide for acrylamide adducts on cysteine. Raw data files are publicly available via the Chorus data repository (https://chorusproject.org) with project I.D. number 1266. Original MaxQuant result files can be provided upon request.

#### RNA interference and transfection

For siRNA experiments, HT-29 cells were seeded at 60% confluence in media without penicillin—streptomycin and allowed to attach overnight. Cells were transfected with 100 nM of *p53* or non-targeted siRNA smart pool from Dharmacon for 6 h using Lipofectamine 2000 (Invitrogen) as per manufacturers protocol. At the end of the incubation period equal volume of McCoy’s media with 40% FBS was added, next morning fresh media with 10% FBS was added and the cells were allowed to grow for 72 h. Cells were harvested by scraping into the media, washed with PBS and lysed for chromatin fractionation.

### *Live cell imaging*

Cells were seeded at 2 × 10^5^ per well in a 12-well glass bottom plate (MatTek, Ashland, MA, USA). Detection of apoptotic cells was performed using the CellEvent™ Caspase 3/7 Green Detection Reagent (Life Technologies). After treatment, cells were stained with 50 µl CellEvent Caspase-3/7 green ready probes reagent and 50 µl ReadyProbes Cell Viability Imaging Kit Blue/Red (Life Technologies) for 15 min at room temp. *z*-stack images of stained cells were taken by confocal microscopy using a Nikon A1 confocal microscope with 20x objective. Active caspase-3/7: green fluorescence, Propidium iodide: red fluorescence, Nuclear DNA: blue fluorescence.

### *In situ PLA*

Cells were seeded at 2 × 10^5^ per well in a 12-well glass bottom plate (MatTek). After removing the media, cells were rinsed with cold PBS three times, fixed in 4% formaldehyde for 15 min and permeabilized in 0.5% Triton x-100 in PBS for 10 min at room temperature. After washing three times in PBS and one time in distilled water for 2 min, cells were then carried out PLA assay using Duolink in situ red kit goat/rabbit (Sigma-Aldrich) according to the manufacturer’s instructions. Briefly, cells were incubated in the blocking buffer for 30 min at 37 °C in a humidified chamber and then incubated with primary antibodies diluted in the antibody diluents overnight at room temperature in a humidified chamber. On the following day, cells were washed in Buffer A for 5 min three times and incubated with the PLA probes (anti-rabbit minus and anti-Goat plus) for 60 min at 37 °C in a humid chamber. This was followed by 5 min wash in Buffer A for two times. The ligation reaction was carried out at 37 °C for 60 min in a humid chamber followed by two times 2 min wash in Buffer A. Cells were then incubated with the amplification mix for 100 min at 37 °C in a darkened humidified chamber. After washing with 1× Buffer B for 10 min for two times and a 1 min wash with 0.01× buffer B, cells were mounted with mounting media containing with 4′,6-diamidino-2-phenylindole (DAPI). *z*-stack images were taken using Nikon A1 confocal microscope with 60× objective oil immersion. The acquisition software is Nikon elements. The primary antibodies were rabbit anti-p53 (Cat# A300-247A) and goat anti-MCM2 (Cat# A300-122A) from Bethyl Laboratories.

### Immunoprecipitation (IP) assays

IP assays were performed as previously described^50^ to determine mtp53 binding proteins in human cancer cells and mouse tumor tissues. In brief, 1 × 10^6^ p53 null H1299 cells were transfected with expression vectors of wtp53 or mutp53 (R175H). Cells were collected and lysed in NP-40 buffer 24 h after transfection for IP experiments by using anti-p53 antibody (DO-1) (Santa Cruz) to pull down mtp53 and its binding proteins. For tissues of normal and thymic lymphomas from mtp53 knock-in mice (Trp53R172H/R172H) as well as thymic lymphomas from p53 knockout mice, 1 mg tissue lystates in NP-40 buffer were used for IP using anti-p53 antibody (FL393) (Santa Cruz).

## Electronic supplementary material

Supplementary Figure 1

Supplementary Data
