# Supplementary figures and images for: Identification, validation, and targeting of the mutant p53-PARP-MCM chromatin axis in triple negative breast cancer
Source: NPJ Breast Cancer. 2017 Jan 19;3:1. doi: 10.1038/s41523-016-0001-7 (PMC5319483; doi:10.1038/s41523-016-0001-7)

**A****shp53 Induction**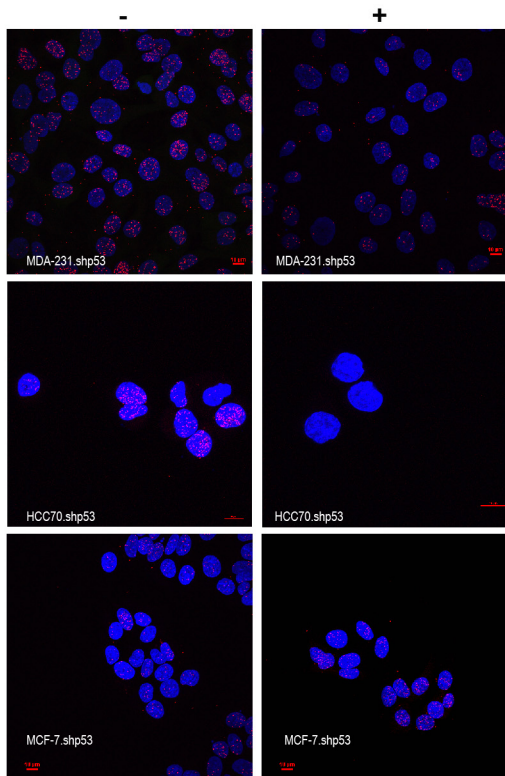**B**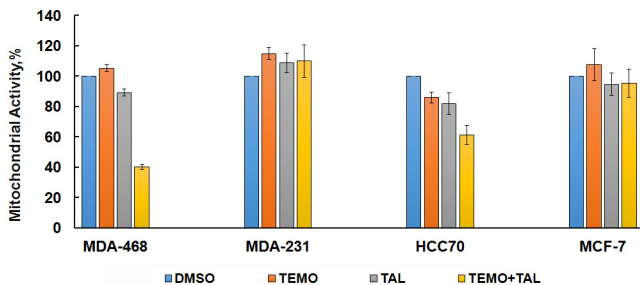

Supplement: Supplementary file 1 — Supplementary Figure 1 [file 41523_2016_1_MOESM1_ESM.pdf]
