# Supplementary material for: Identification, validation, and targeting of the mutant p53-PARP-MCM chromatin axis in triple negative breast cancer
Source: NPJ Breast Cancer. 2017 Jan 19;3:1. doi: 10.1038/s41523-016-0001-7 (PMC5319483; doi:10.1038/s41523-016-0001-7)
Supplement: Supplementary file 2 — Supplementary Data [file 41523_2016_1_MOESM2_ESM.docx]

S1:  **Mutant p53 R280K and R248Q associate with MCM2.** (A) Analysis of p53/MCM2 complexes (red) by immunofluorescence microscopy in combination with *in situ* proximity ligation assay (PLA) in MDA-231.shp53, HCC70.shp53 or MCF.shp53 cells grown in the presence or absence of doxycycline for 7d. DNA was counterstained with DAPI (blue). The z stack confocal maximum intensity projection images of p53/MCM2 and DAPI are shown. Doxycycline concentration used in each cell line was titrated to knockdown p53: MDA-231.shp53: 8μg/ml; HCC70.shp53: 6μg/ml and MCF7.shp53: 2μg/ml. (B) Mitochondrial activity was measured after treatment of 1mM temozolomide (Temo), 10μM talazoparib (Tal) or combination of Temo + Tal (Temo +Tal) in MDA-468, MDA-231, HCC70 or MCF7 cells for 24hrs. MTT assay shows significant reduction of mitochondrial activity after 24 hrs of combination treatment in MDA-468 cells, no effect in MDA-231 and MCF-7 and some decrease in HCC70 cells.
